# Hepatitis B and C: prevalence and risk factors associated with seropositivity among children in Karachi, Pakistan

**DOI:** 10.1186/1471-2334-6-101

**Published:** 2006-06-23

**Authors:** Wasim Jafri, Nadim Jafri, Javed Yakoob, Muhammad Islam, Syed Farhan Ali Tirmizi, Tazeen Jafar, Saeed Akhtar, Saeed Hamid, Hasnain Ali Shah, Sheikh Qamaruddin Nizami

**Affiliations:** 1 Department of Medicine, Aga Khan University Hospital, Karachi, Pakistan

## Abstract

**Background:**

Infections with hepatitis B virus (HBV) and hepatitis C virus (HCV) can lead to chronic liver disease and hepato-cellular carcinoma (HCC). This cross-sectional study estimated the prevalence and identified risk factors associated with Hepatitis B surface antigen (HBsAg) and HCV antibody (anti-HCV) sero-positivity among children 1 to 15 years of age.

**Methods:**

The study targeted the low to middle socioeconomic population that comprises 80% to 85% of the population. Consent was obtained from parents of the eligible children before administering questionnaire and collected a blood sample for anti-HCV and HBsAg serology.

**Results:**

3533 children were screened for HBsAg and anti-HCV. 1826 (52 %) were males. 65 (1.8 %) were positive for HBsAg, male to female ratio 38:27; mean age 10 ± 4 years. 55 (1.6 %) were positive for anti-HCV with a mean age 9 ± 4 years. 3 (0.11%) boys were positive for both HBsAg and anti-HCV. The overall infection rate was 3.3 % in the studied population. Hepatitis BsAg was more prevalent in subjects who received therapeutic injections 45 (69.2%) positive [Odd Ratio OR = 2.2; 95% Confidence interval CI: 1.3–3.6] inspite of using new needle and syringe 44 (67.7%) positive [OR = 2.2; 95% CI: 1.3–3.7] and vaccination in the government healthcare facilities 46 (70.7 %) positive with [OR = 3.0; 95% CI: 1.4–6.4]. These factors were not significant in anti-HCV positive cases.

**Conclusion:**

There is a need to educate general population regarding HBV and HCV infection and risks associated with inappropriate therapeutic injections. Hepatitis B vaccine should be administered to all newborns regardless of maternal HBsAg status.

## Background

Viral hepatitis is a major public health problem in all parts of the world. Pakistan is in the intermediate HBV prevalence area with a carrier rate of 3–4% [1]. Chronic hepatitis B is a severe problem in Pakistan [2,3]. In a community based study 31% percent had hepatitis B core antibodies and 4.3% had hepatitis B surface antigen [4]. In an earlier study the frequency of HBsAg in healthy subjects was 2.9% and HBs Ab 35% [5]. Horizontal transmission, particularly in early childhood, accounts for most cases of chronic HBV infection in intermediate prevalence areas like Pakistan [6]. Children may acquire HBV infection through horizontal transmission via minor breaks in the skin or mucous membranes or close bodily contacts with other children. Zuberi et al. described HBsAg prevalence of 2.5% in pregnant women and out of these 17% HBeAg and 61% anti HBe positive [7]. Low frequency of HBsAg and HBeAg in pregnant women makes vertical transmission a less important cause of transmission [8].

The burden of HCV related chronic liver disease (CLD) in Pakistan has increased. Earlier studies showed that of all patients presenting with CLD, 16.6% were anti-HCV positive [9] more recent data shows nearly 60–70% patients with CLD tend to be positive for anti-HCV [2,10,11]. It has been demonstrated that nearly 50% patients with hepatocellular carcinoma (HCC) in Pakistan are anti-HCV positive [12]. Blood transfusions is still the major cause of HCV transmission in the country; as a survey of blood banks in the large urban centers of the country showed that only about 25% of them tested blood and blood product donations for HCV infection to keep the cost down. [13]. A number of studies have shown the relationship between therapeutic injections using non-sterile needles and the transmission of HCV [14]. There is an enormous dependence on parenteral therapy for treatment, both in the form of injections and infusion of drips, driven by cultural beliefs in the power of parenteral therapy. Additional risk factors that may be important modes of transmission include are excessive use of barbers for shaving [15], ear piercing and non-sterile surgical and dental practices of unqualified health care workers (quacks).

The mean age of developing CLD in developing countries including Pakistan is much lower as compared to developed countries, suggesting that individuals are being infected at a younger age in this part of the world. In 1994, a cross sectional study done in children revealed 3% were HBsAg positive [16]. The sero-prevalence of HCV in children appears to be low in Pakistan, with 0.2% and 0.4% children infected under the age of 12 and between 12–19 years respectively [16]. The aim of this study was to estimate the prevalence of anti-HCV and HBsAg to screen for current infection and also to identify the risk factors associated with anti-HCV and HBsAg sero-positivity among children (1 to 15 years of age) in Karachi, Pakistan.

## Methods

### Study design and setting

The study was carried out in May, 2003- August, 2004 and targeted the low to middle socioeconomic population of Karachi which constitute about 80% to 85% of the population. Also, this group is less educated and more exposed to some of the important established risk factors for the diseases under study. All children 1 to 15 years of age living in the study were eligible to participate in the survey. A prospective cross-sectional study was conducted to achieve the primary objective to estimate the prevalence of anti-HCV and HBsAg sero-positivity and evaluate potential risk factors by comparing seropositive and seronegative for HBsAg and anti-HCV antibody among children 1 to 15 years of age. A two-stage cluster sampling technique was employed to draw the required sample. The Federal Bureau of Statistics (FBS) divided the city into 5000 clusters each of about 250 households. On average each household contained 7 individuals of which about 40% (3 individuals per household) were aged 1 to 15 years. About 85% (4250) of the total 5000 clusters were in the lower and middle-income group (average household income less than Rupees 7440/month). The survey was conducted in ten geographically separate clusters. Sampling unit was a household selected from within each cluster through random sampling. We randomly selected about 338 children from each sampled cluster that contained about 700 children 1 to 15 years of age.

### Data collection and serology

Trained community health workers (CHW) reached the selected household and approached the head of the family. They explained the purpose and objectives of the study and asked for written informed consent from parents of the eligible children before administering the questionnaire and collecting a blood sample. The trained CHW administered a pre-tested questionnaire to the mother/father of each child to obtain information on socio-demography and potential risk factors for HCV and HBV transmission after taking written informed consent. The questions in the questionnaire gathered following information in addition to demographic characteristics including age, gender, socio-economic status, educational level in years, number of injections in past six months to ten years, type of health care provider for injections. History of surgical procedure, especially circumcision in male children, blood or blood products transfusion in child, factors predisposing to horizontal transmission of Hepatitis B, history or current use of intravenous drugs in the child, siblings or in other family members; history of tattooing or ear piercing in child; jaundice or diagnosis of HCV or HBV among siblings, parents or other family members living in the same house. The collected samples were centrifuged within 6 hours of collection and after serum separation stored at -70°C. Serology for anti-HCV and HBsAg was performed on the collected blood samples after the completion of data collection. Third generation enzyme linked immunosorbent assay (ELISA) visual test kits for anti-HCV and HBsAg (by International Immuno Diagnostics CA, USA) were used to detect the presence of anti-HCV and HBsAg in the sera of the study subjects.

### Data management and analysis plan

EPI Info version 6 was used for data entry [17]. The data was analyzed using Statistical Package for Social Sciences (SPSS) version 10.0 [18]. The analysis was carried out at three levels descriptive analysis, univariate and multivariate analysis. Descriptive statistics of socio-demographic variables and other characteristics of the sampled population were computed. Means and standard deviations (SD) were calculated for quantitative variables and proportions for categorical variables. Univariable logistic regression analysis was performed to measure association of outcome with each independent variable. Odds ratios (OR) and 95% confidence intervals (CI) was calculated for each association. Associations among independent variables were assessed using appropriate tests before performing multivariate analysis. Multiple logistic regression models were used to examine the association between the independent variables and the main outcome variables, Hepatitis B and C virus, while controlling for the effects of other covariates. P value < 0.05 was considered as statistical significant.

Using software EPI Info [17] and assuming 4% prevalence of anti-HCV in the study population with 95% confidence level and a bound on error of +1 % the estimated sample size was 1475. Sample size required for the ratio of children with HCV and children without HCV of 1: 6, to detect an odds ratio of 3.0 at 95% confidence level and power of 80%, for the prevalence of risk factor estimated to be 6% was 1617 children using the EPI Info software [17]. Sample size calculated for the risk factor was larger than the one for estimation of prevalence of HCV. Therefore, a sample of 1617 children was targeted and this sample size covered the primary and the secondary objectives. For cluster sampling we incorporated the design effect. We calculated a design effect of 1.9 [19]. The sample size required after multiplying with design effect is: n = 1617 × D = 1617 × 1.9 = 3073. However, this sample size was inflated by 10%, to account for non-responders, making the final target sample size of 3380 children.

### Ethical issues

Informed written consent was obtained from the parent of the study subject. The serological results were provided to the parents at the completion and kept strictly confidential. The parents of infected children were provided with appropriate information on the prevention of further spread of these infections and referred to the nearest government medical facility for guidance.

## Results

All the parents were interviewed and a total of 3533 children were screened for HBsAg and anti-HCV. The age range was 1–15 years. 1826 (52%) were males and 1707 (48%) were females. The over all infection rate was 3.3 in the studied population. 65 (1.8%) was positive for HBsAg with male to female ratio of 38:27 (Table 1). Those positive for HBsAg had a mean age of 10 ± 4 years. 55 (1.6%) was positive for anti-HCV with a mean age of 9 ± 4 years. 32 (58%) were boys and 23 (42%) were girls (Table 2). 3 (0.11%) was positive for both HBsAg and anti-HCV and they were all males.

**Table 1:** Demographic features and risk factors associated with seroprevalence of Hepatitis B in children

	**Hepatitis B virus**			
			
**Risk Factor**	**Positive n (%)**	**Negative n (%)**	**Odds Ratio (OR) 95% confidence interval (CI)**	**P value**
**Therapeutic Injections**				
Yes	45 (69.2)	1766 (50.9)		
No	20 (30.8)	1702 (49.1)	2.2 (1.3 – 3.6)	0.003

**Use of new needle & and syringe**				
Yes	44 (67.7)	1694 (48.8)		
No	21 (32.3)	1774 (51.2)	2.2 (1.3 – 3.7)	0.003

**No: of Injections in the last year**				
<2 injections	4 (12.5)	371 (28.2)	Reference	
2–5 injections	13 (40.7)	462 (35.2)	2.6 (0.8 – 8.1)	
>5 injections	15 (46.8)	481 (36.6)	2.9 (1.0 – 852)	0.140

**Received Blood Transfusion**				
Yes	1 (1.5)	51 (1.5)		
No	64 (98.5)	3417 (98.5)	1.0 (0.1 – 7.7)	0.964

**Hospitalized**				
Yes	16 (24.6)	590 (17)		
No	49 (75.4)	2878(83)	1.6 (0.9 – 12.8)	0.107

**Family member died of Liver disease**				
Yes	7(10.8)	361 (10.4)		
No	58 (89.2)	3107 (89.6)	1.0 (0.5 – 2.3)	0.925

**Diagnosis in Family member**				
HCV	1 (14.3)	12 (3.2)		
HBV	1 (14.3)	36 (10)	-	
Jaundice	5 (71.4)	306 (84.8)		
Liver disease	0	7 (2)		0.433

**Sharing of various items with infected person**				
Yes	4 (57.1)	110 (31)		
No	3 (42.9)	244 (69)	3.0 (0.7 – 13.4)	0.147

**Previous Immunization**				
Vaccinated	54 (83)	3001(86.5)		
Non-Vaccinated	11 (7)	467 (13.5)	1.3 (0.7 – 2.5)	0.419

**Place vaccinated**				
Private Sector	7 (10.8)	1030 (29.7)	Reference	
Govt. Sector	46 (70.7)	1974 (57)	3.4 (1.5 – 7.6)	
None	12 (18.5)	464 (13.3)	3.8 (1.5 – 9.7)	0.022

**Visit to Barber Shop**				
Yes	47 (72.3)	2814 (81.1)		
No	18 (27.7)	654 (18.9)	0.6 (0.4 – 1.1)	0.072

**Received cuts at Barber Shop**				
Yes	11 (23.4)	686 (24.4)		
No	36 (76.6)	2128 (75.6)	0.9 (0.5 – 1.9)	0.877

**Child's ear/nose pierced**				
Yes	22 (33.8)	1293 (37.3)		
No	43 (66.2)	2175 (62.7)	0.9 (0.5 – 1.4)	0.570

**Dental treatment**				
Yes	0	107 (3)		
No	65 (100)	3361 (97)	-	0.150

**Father education status**				
Educated	34 (52.3)	2300 (66.3)		
Non-Educated	31 (47.7)	1168 (33.7)	1.8 (1.1 – 2.9)	0.018

**Mother education status**				
Educated	25 (38.5)	1964 (56.6)		
Non-Educated	40 (61.5)	1504 (43.4)	2.1 (1.3 – 3.5)	0.003

**Ethnic Origin**				
Sindhi	1 (1.5)	86 (2.5)	Reference	
Balochi	12 (18.5)	133 (3.9)	7.8 (1.0 – 60.8)	
Punjabi	10 (15.4)	996 (28.8)	0.9 (0.1 – 6.8)	< 0.001
Pathan	4 (6.2)	179 (5)	1.9 (0.2 – 17.5)	
Mohajirs	19 (29.2)	1796 (51.8)	0.9 (0.1 – 6.9)	
Others	19 (29.2)	278 (8)	5.3 (0.7 – 40.1)	

**Age Group (years)**				
≤ 5 years	17 (26.2)	915 (26.4)	Reference	
6 – 9 years	14 (21.5)	931 (26.8)	0.8 (0.4 – 1.7)	
10 – 13 years	16 (24.6)	934 (27)	0.9 (0.5 – 1.8)	
> 13 years	18 (27.7)	688 (19.8)	1.4 (0.7 – 2.8)	0.428

**Gender**				
Boys	38 (58.5)	1788 (51.6)		
Girls	27 (41.5)	1680 (48.4)	1.3 (0.8 – 2.2)	0.270

**Table 2:** Demographic features and risk factors associated with seroprevalence of anti-Hepatitis C in children

	**Anti-Hepatitis C antibody**			
			
**Risk Factor**	**Positive n (%)**	**Negative n (%)**	**Odds Ratio (OR) 95% confidence interval (CI)**	**P value**
**Therapeutic Injections**				
Yes	28 (50.9)	1783 (51.3)		
No	27 (49.1)	1695 (48.7)	0.9 (0.6 – 1.6)	0.958

**Use of new needle & and syringe**				
Yes	28 (50.9)	1710 (49.2)		
No	27 (49.1)	1768 (50.8)	1.0 (0.6 – 1.8)	0.798

**No: of Injections in the last year**				
<2 injections	7(38.9)	368 (27.7)	Reference	
2–5 injections	4 (22.2)	471 (35.5)	0.4 (0.1 – 1.5)	
>5 injections	7 (38.9)	489 (36.8)	0.8 (0.3 – 2.2)	0.427

**Received Blood Transfusion**				
Yes	2 (3.7)	50 (1.4)		
No	53 (96.3)	3428 (98.6)	2.6 (0.6 – 10.9)	0.179

**Hospitalized**				
Yes	12 (21.8)	594 (17)		
No	43 (78.2)	2884(83)	1.4 (0.7 – 2.6)	0.355

**Family member died of Liver disease**				
Yes	8(14.5)	360 (10.3)		
No	47 (85.5)	3118 (89.7)	1.5 (0.7 – 3.1)	0.312

**Diagnosis in Family member**				
HCV	1 (12.5)	12 (3.4)		
HBV	0	37 (10.5)	-	
Jaundice	7(87.5)	304 (86.1)		0.380

**Previous Immunization**				
Vaccinated	47 (85.5)	3008 (86.5)		
Non-Vaccinated	8 (14.5)	470 (13.5)	0.9 (0.4 – 2.0)	0.842

**Place vaccinated**				
Private Sector	14 (25.5)	1023 (29.4)	Reference	
Govt. Sector	33 (60)	1987 (57.1)	1.2 (0.6 – 2.3)	
None	8 (14.5)	469(13.5)	1.2 (0.5 – 3.0)	0.930

**Visit to Barber Shop**				
Yes	45 (81.8)	2816 (81)		
No	10 (18.2)	662 (19)	1.1 (0.5 – 2.1)	0.873

**Received cuts at Barber Shop**				
Yes	7 (15.6)	690 (24.5)		
No	38 (84.4)	2126 (75.5)	0.6 (0.3 – 1.3)	0.165

**Child's ear/nose pierced**				
Yes	19 (34.6)	1296 (37.2)		
No	36 (65.4)	2182 (62.8)	0.9 (0.5 – 1.6)	0.679

**Dental treatment**				
Yes	1 (1.9)	106 (3)		
No	54 (98.1)	3372 (97)	0.6 (0.1 – 4.3)	0.598

**Father education status**				
Educated	36 (65.5)	2298 (66)		
Non-Educated	19 (34.5)	1180 (34)	1.0 (0.6 – 1.8)	0.924

**Mother education status**				
Educated	24 (43.7)	1965 (56.5)		
Non-Educated	31 (56.3)	1513 (43.5)	1.7 (0.9 – 2.9)	0.056

**Ethnic Origin**				
Sindhi	1 (1.8)	86 (2.5)	Reference	
Balochi	3 (5.5)	142 (4)	1.8 (0.2 – 17.7)	
Punjabi	16 (29)	990 (28.5)	1.4 (0.2 – 10.6)	
Pathan	4 (7.3)	179 (5.1)	1.9 (0.2 – 17.5)	0.568
Muhajirs	23 (41.8)	1792 (51.5)	1.1 (0.1 – 8.3)	
Others	8 (14.6)	289 (8.4)	2.4 (0.3 – 19.3)	

**Age Group (years)**				
<= 5	13 (23.5)	919 (26.4)	Reference	
6–9	14 (25.5)	931 (26.8)	1.1 (0.5 – 2.3)	
10–13	14 (25.5)	936 (27)	1.1 (0.5 – 2.3)	
> 13	14 (25.5)	692 (19.8)	1.4 (0.7 – 3.1)	0.783

**Gender**				
Boys	32 (58.2)	1794 (51.6)		
Girls	23 (41.8)	1684 (48.4)	0.7 (0.4–1.3)	0.331

### Demographic features of the study population

#### Area of study population

The distribution of study population was 1328 (37.6%) in the north, 443 (12.5%) west, 439 (12.4 %) east, 878 (24.9%) south and 445 (12.6%) central area of the city.

In reference to other areas i.e. 18 (1.4%) north and 3 (0.7%) west of the city where overall HBsAg prevalence was 21 (2.1%), the prevalence of HBsAg in the east was 24 (5.5%) [OR = 4.8; CI: 2.7–8.7 p < 0.001] and in south 18 (2.1 %) [OR = 1.8; CI: 0.3–3.3 p = 0.08]. The distribution of anti-HCV positive cases was 8 (1.8%) in the east, 11 (2.5%) central, 16 (1.8 %) south, 17 (1.3%) and west 3 (0.7%). However, anti-HCV distribution was not statistically significant among different areas of the city.

#### Accommodation

The living quarters of the studied population both HBsAg and anti-HCV positive and negative had a mean of 2 rooms (range of 1–7 rooms). It was being shared among 7 individual household members.

#### Educational status

The educational status of the parents showed that when mothers were uneducated it was significantly associated with positive HBsAg status on Univariable analysis (Table 1) however, it was not on multivariable analysis (Table 3). Educational status of the parents was not significantly associated with anti-HCV positive (Table 2).

**Table 3:** Independent risk factors associated with seroprevalence of Hepatitis B in children identified in multiple logistic regression

**Risk Factor**	**Adjusted odds Ratio (AOR)**	**95% confidence interval (CI)**	**P value**
**Use of new needle & and syringe**			
No	Reference		
Yes	1.9	(1.1 – 3.3)	0.033

**Place vaccinated**			
Private Sector	Reference		
Govt. Sector	2.4	(1.1 – 5.2)	0.030

**Father education status**			
Educated	Reference		
Non-Educated	1.4	(0.7 – 2.7)	0.333

**Mother education status**			
Educated	Reference		
Non-Educated	1.4	(0.6 – 2.7)	0.361

#### Ethnic origin

The ethnic distribution of various groups in the study population is shown in Table 1, 2. 19 (29.2 %) of the children positive for HBsAg were ethnic mohajirs, 10 (15.4 %) punjabi, 4 (6.2 %) pathan, 12 (18.5 %) balochi, 1 (1.5%) sindhi while 19 (29.2%) belonged to other ethnic groups. 23 (41.8 %) mohajirs, 16 (29 %) punjabi, 4 (7.3 %) pathan, 3 (5.5 %) balochi, 1 (1.8 %) sindhi and others 8 (14.6 %) were positive for anti-HCV. HBsAg and anti-HCV positivity did not demonstrate statistically significant distribution in various ethnic groups.

#### Occupation of parents

27 (42%) of the HBsAg and 39 (71%) of the anti-HCV positives father were employed as daily wage earners while 60 (92%) of the HBsAg and 52 (94%) of anti-HCV mothers were housewives.

#### Prior knowledge regarding Hepatitis B and C

60 (92%) HBsAg positive patients parents have not heard of Hepatitis B (p = 0.830). 51 (93%) anti-HCV positive patients parents have not heard of Hepatitis C virus (p = 0.815).

### Risk factors

#### Therapeutic injections

45 (69.2%) children with HBsAg received therapeutic injection as compared to 1766 (50.9%) HBsAg negative p = 0.003 (Table 1). 28 (50.9%) with anti-HCV received therapeutic injections as compared to anti-HCV negative 1783 (51.3%) p = 0.958 (Table 2). 44 (67.7%) HBsAg positive received therapeutic injection with a new syringe and needle as compared to 1694 (48.8%) HBsAg negative p = 0.003 (Table 1). 28 (50.9 %) anti-HCV claimed to be given by a new plastic syringe and needle every time as compared to anti-HCV negative 1710 (49.2%) with [OR = 1.10; CI: 0.6–1.8 p = 0.798] (Table 2). There was an increase in the number of HBsAg positive with an increase in the number of therapeutic injections p = 0.140 (Table 1) however, no such relationship was demonstrated with anti-HCV p = 0.427 (Table 2).

#### Previous immunization

54 (83%) HBsAg positive received vaccination while 3001(86.5%) did not (Table 1). Of those vaccinated 46 (70.7 %) received vaccination in the government healthcare facilities while only 7 (10.8%) in the private sector [OR = 3.4; CI: 1.5–7.6 p = 0.02] (Table 1). 47 (85.5 %) anti-HCV positive received vaccination while 8 (14.5 %) did not. Of those vaccinated 33 (60%) received vaccination in the government healthcare facilities while only 14 (25.5%) in the private sector p = 0.93 (Table 2). Children with previous vaccination in the government sector were significantly positive for HBsAg 46 (70.7%) with [OR = 3.4; CI: 1.5–7.6 p = 0.02] (Table 1). However, this was not so for anti-HCV (Table 2). There was no history of a child with HBsAg or anti-HCV positive ever been pricked accidentally by a used syringe and needle.

#### Visit to the Barber in those with positive serology

47 (72.3%) children with HBsAg p = 0.07 (Table 1) and 45 (81.8%) with anti-HCV p = 0.873 visited a barber for a haircut (Table 2). 11 (23.4%) children with HBsAg p = 0.8 (Table 1) and 7 (15.6%) with anti-HCV p = 0.6 received accidental cuts at the barber for a haircut (Table 2).

#### Piercing of nose and ears in those with positive serology

22 (33.8%) HBsAg positive p = 0.570 and 19 (34.6%) anti-HCV positive p = 0.679 had piercing of nose or ears (Table 1, 2).

#### Dental treatment

Previous dental treatment did not feature in HBsAg positive while 1 (0.9 %) with anti-HCV positive received it (Table 2).

#### Tattooing

Tattooing was not practiced by the positive patients and there was also no history of intravenous or other form of drug addiction.

### Discussion and conclusion

Pakistan lies between middle to low income countries with over one-twelfth of labor force is unemployed, over one third of the population subsists in poverty and over half the population is illiterate, with parts of the country being worse than what the national average indicates [20]. In 2001–2002 Pakistan received a grant from the Global Alliance for Vaccines and Immunization (GAVI) that has enabled the introduction of Hepatitis B vaccination in routine Expanded Program on Immunization (EPI) [21]. Vaccination for HBV as part of EPI was launched in a nationwide vaccination campaign in 2004 [22]. Special attention was given to children under 1 year of age and to married women who might bear children.

The limitations of our study are that we did not check the hepatitis B core antibody and liver function tests. It would have increased the cost and limited the study to a comparatively smaller size. This has led us to assess the extent of problem in our vulnerable population and it will help our health policy maker in future planning. In our study, the prevalence of HBsAg was 1.8 percent and anti-HCV 1.6 percent in children with a mean age of 10 years and 9 years, respectively. These children have received primary schooling but their parents were mostly uneducated. The educational status of parents of HBsAg positive children was significantly low only on univariable analysis (Table 1, 3). Both children and their parents had a poor knowledge of HBV and HCV related diseases. A number of HBV positive children received therapeutic injections from local general practitioners not using a new syringe. However, children receiving therapeutic injections with new needles and syringes also demonstrated a high prevalence of HBV. This could be explained by unlawful practices in the country of used syringes being washed and packaged for re-sale [23]. This highlights the inadequate system of medical waste disposal in the community. To maximize their profit medical practitioners in the private sector routinely reuses syringes and only changes the needle when it gets blunt. It is estimated that about half of all injections administered in Pakistan involve reused syringes [23]. Also, use of multiple-dose vials is considered an important source of patient-patient transmission of hepatitis B and C. In our study, number of cases with HBV increased with the number of injections received. However, this was not observed in children with positive HCV. In an earlier study, non-sterile syringes and needles were also the source of HCV and HBV infections in a peri urban community of Karachi [24]. Patients who received more injections were more likely to be infected with HCV [24]. Also, non-sterile syringes and needles that had been used earlier in the day on other patients were used for 94% of the observed injections. Previous hospitalization, dental treatment, blood transfusion, visiting and receiving cuts at Barber shops, piercing of ears and nose, presence of HBsAg carriers and/or HCV chronically infected subjects among family members, death of a family member due to liver disease did not feature as contributing risk factors in our study. There was no history of needle stick injuries and practice of tattooing. Piercing of nose and ears, use of inadequately sterilized instruments, with clients receiving accidental cuts in various practices including in barber shops were reported. Previous vaccination was largely received at the public health-care facilities. A nationwide survey by the ministry of health in 2002 revealed that as many as 72% therapeutic injections and 50% immunization injections in public health-care facilities were unsafe and potentially dangerous [23]. The irrational use of drugs is a major problem. When prescriptions from 60 public and 48 private health facilities were compared over 48% of GP prescriptions had at least one injectable drug compared with 22.0% by public providers (*p *< 0.0001) [25].

The implication of this study is that seropositivity was comparatively higher for HBsAg in children than for anti-HCV. However, the difference between their prevalence was not statistically significant. This suggests that mother/child transmission of HBV may have also followed. The possible contribution to prevalence of HBsAg of a birth to an HBV carrier mother cannot be ruled out. However, HBsAg and anti-HCV status of mothers was not established in this study. It is difficult to estimate what percent of these viral infections could be attributed to each of the risk factors in our study as numbers of positive cases were small and in HCV positive cases none achieved even univariable significant level. In a previous study, eight (3.3%) of 245 mothers tested positive for HBsAg, all babies born to mothers with HBV infection were negative for this marker [26,27]. It concluded that vertical transmission in the early perinatal period is the least. In comparison perinatal transmission of HCV occurs at the time of birth in about 5 percent of infants born to anti-HCV positive women almost exclusively from mothers who are HCV-RNA positive [28,29]. General sero-prevalence of anti-HCV and HBsAg in healthy adult male population from the northern Pakistan was 5 percent for anti-HCV and 2.5 percent for HBsAg [30]. This reflects that the population at large is exposed to the same risk factors. Low educational level and/or low socio-economic status has also been associated with the prevalence of a number of infectious diseases. This is in keeping with Mujeeb et al who described areas of higher prevalence of Hepatitis C located in specific districts and a trend of higher prevalence in less affluent urban areas for the city of Karachi [31]. In our study the odd of a positive test for HBsAg was 2.2 in those receiving therapeutic injection as compared to those who did not. It is imperative that for eradication of HBV infection universal vaccination of all newborns is carried out together with education of the public to limit injections to those which are safe and clinically indicated.

## Competing interests

The author(s) declare that they have no competing interests.

## Authors' contributions

WJ conceived the idea; WJ, NJ, JY, MI, SFAT contributed to the study data interpretation; WJ, NJ, JY wrote the paper. TJ, SA, SH, HAS, SQN contributed to the study and critically analyzed the manuscript.

## Pre-publication history

The pre-publication history for this paper can be accessed here:


